# Coronary artery calcium scoring as a tool for risk stratification in patients with an elevated lipoprotein(a) level

**DOI:** 10.3389/fcvm.2022.1084814

**Published:** 2022-12-22

**Authors:** Robert Naami, Drew M. Miller, Sadeer Al-Kindi, Ian J. Neeland

**Affiliations:** ^1^School of Medicine, Case Western Reserve University, Cleveland, OH, United States; ^2^University Hospitals Cleveland Medical Center, Cleveland, OH, United States; ^3^Center for Cardiovascular Prevention, Harrington Heart and Vascular Institute, Cleveland, OH, United States

**Keywords:** coronary artery calcification (CAC), lipoprotein(a), atherosclerotic vascular disease, risk stratification, prevention

Genetic and epidemiologic studies have demonstrated that elevated serum levels of Lp(a) increase risk for atherosclerotic vascular disease (ASCVD), with guidelines suggesting that levels ≥50 mg/dL or ≥125 nmol/L be used as a risk-enhancing factor when adjudicating ASCVD risk (1). However, several obstacles have limited routine Lp(a) assessment and result application in clinical practice. These include the lack of a standardized quantification method, variable expression among different races and ethnicities, and the lack of clear guidance when to treat along with limited therapeutic options when an elevated test result is received. There is a need for a more targeted approach that goes beyond contemporary risk stratification paradigms with the goal to be more aggressive at implementing known risk reducing therapies, especially among younger individuals with elevated risk [e.g., high Lp(a) and family history of ASCVD] who might not otherwise meet standard indications for statin and other therapies. One strategy would be to link Lp(a) testing with an established tool for assessing ASCVD risk, known to improve risk discrimination. Coronary artery calcium scoring (CAC) is an ideal candidate given its increasingly prevalent use, previously shown association with Lp(a), and the potential benefits of a dual risk stratification system (2–5).

To better understand the potential utility of CAC and Lp(a) for ASCVD risk, it is important to characterize the individual and joint associations that CAC and Lp(a) have with ASCVD incidence in diverse populations (Table 1). Given the varying pathophysiology between CAC and Lp(a), each test may contribute independent, and potentially synergistic, information for risk assessment. Lp(a) promotes atherogenesis and thrombosis with levels genetically determined and unaffected by diet, exercise, and use of statins. Conversely, CAC is a measure of atheroma calcium content that correlates with plaque burden and is directly related to traditional atherosclerotic risk factors such as blood pressure, weight, and serum cholesterols. In a study of the MESA and DHS cohorts, Mehta et al. (6) recently showed that elevated Lp(a) level and CAC score were each independently associated with ASCVD incidence. Moreover, among participants with elevated CAC (i.e., ≥100 AU), the top Lp(a) quintile (as stratified by race) was associated with an ASCVD event rate of >20% while the same elevated Lp(a) levels had ASCVD event rate of < 10% when coupled with CAC < 100. This was following adjustments for traditional risk factors and family history of heart disease (6). This suggests one of two possibilities. First is that CAC scoring can help inform care among patients with elevated Lp(a). Alternatively, elevated Lp(a) further potentiates elevated risk due to a high CAC score. This reinforces the current standard of practice where Lp(a) levels are adjudicated in individuals with strong predisposition for atherosclerotic disease. This includes those with strong family history of cardiovascular disease, repeat events despite appropriate therapy, and following a premature atherosclerotic event. Importantly, the association of Lp(a) with CAC is corroborated by additional studies illustrating a positive correlation (7). In an analysis of German participants without known coronary artery disease, Lp(a) levels and CAC were significantly associated despite adjusting for risk factors such as age, sex, smoking, BMI, lipids, diabetes, antihypertensive medication and lipid-lowering medication. However, it is noteworthy in this study that genetic variants responsible for elevated Lp(a) levels were not associated with CAC scores. This finding supports an independent pathophysiology for Lp(a) such that the burden of calcific disease is not necessarily indicative of long-term exposure to the oxidative and atherothrombotic properties of Lp(a) (8). Further evidence of heterogeneity of effects between CAC and Lp(a) come from a study by Qasim et al. that demonstrated Lp(a) to be a strong predictor of CAC in women with type 2 diabetes but not in men or those without diabetes (9). Explanations for this finding could include a cardioprotective effect of decreased estrogen levels in the postmenopausal female cohort studied; alternatively, Lp(a) function and atherogenicity may be modified by glycation in the milieu of diabetes. Nevertheless, the aforementioned studies did not explore rates of ASCVD incidence and therefore make it challenging to infer meaningful information to help guide clinical management. As such, outcomes driven data are required to fully understand the impact of joint Lp(a) and CAC testing on ASCVD events.

**Table 1:** Summary of contemporary evidence for the relationship between lipoprotein(a) and coronary artery calcium.

**References**	**Study population**	**Variables described in study**	**CAC correlation with Lp(a)**	**Statistical result**
Kullo et al. (4)	GENOA (genetic epidemiology network of Arteriopathy, *n =* 765)	CAC score correlation with Lp(a) quintile	No significant correlation	No significant positive or negative correlation
Pechlivanis et al. (8)	3,800 German patients without CAD	CAC score increase per 1 log-unit increase in Lp(a)	Positive	0.27 (95% CI 0.11–0.44)
Grief et al. (7)	1,600 European patients with typical or atypical chest pain	CAC scores in patients with Lp(a) > 30 and Lp(a) < 30	Positive	CAC 261 ± 201 when Lp(a) > 30 CAC 194 ± 133 when Lp(a) < 30
Qasim et al. (9)	Penn diabetes heart study [*n =* 1,299] and the study of inherited risk of coronary atherosclerosis [*n =* 860]	CAC increase compared with one-unit increase in Lp(a) as a linear relationship	Mixed, positive in diabetic women but no correlation in diabetic men and non-diabetics	2.25 (95% CI 1.34–3.79) in diabetic women
Mehta et al. (6)	MESA (Multi-Ethnic Study Of Atherosclerosis, *n =* 4,512) and DHS (Dallas Heart Study, *n =* 2,078) cohorts	ASCVD events *via* hazard ratio with elevated Lp(a) and CAC	Positive	4.71 (95% CI 3.01–7.40) in elevated Lp(a) and CAC ≥ 100

Further research is needed to define a potential role for joint testing of CAC and Lp(a) and how these tests could be selectively utilized in clinical practice to maximize the risk/benefit ratio and define a high-risk individual for aggressive preventive therapies. Based on the current evidence, it could be argued that there is less utility in pursuing Lp(a) levels without prior evidence of atherosclerotic burden. Alternatively, it raises questions as to whether CAC scoring should precede Lp(a) levels, acting as a gateway to Lp(a) testing, given the high likelihood that an elevated Lp(a) result in that context would prompt more aggressive preventive therapy. To address this gap in knowledge and practice, a prospective study is required to potentially expand our current indications for primary prevention. In particular, we must assess the ASCVD event rate for subjects with Lp(a) cutoff >50 mg/dL and CAC >100 who are without other common risk factors for atherosclerotic disease (i.e., hypertension, tobacco use, etc.). Additionally, with the advent of novel Lp(a) lowering therapies poised to hit the market soon, should there be consideration to add a Lp(a) lowering therapy in addition to statins in individuals with elevated CAC and Lp(a) given the high-risk phenotype and the lack of response of Lp(a) levels to conventional statin therapy? At this time, we lack medications that are proven to lower Lp(a) and provide more favorable cardiovascular outcomes. Niacin, for instance, reduces lipoprotein(a) serum levels by approximately 20% but does not reduce death or ischemic cardiovascular events (10, 11). Additionally, in a *post-hoc* analysis of the FOURIER study evaluating PCSK9 inhibition (PCSK9i), PCSK9i-treated groups incurred an estimated 20 mg/dL absolute reduction in Lp(a) associated with a 10% relative risk reduction in MACE (12). Nevertheless, we have not completed prospective clinical studies to corroborate these findings. For now, the cardiovascular community eagerly awaits the results of ongoing trials (i.e., HORIZON) investigating the impact of dedicated Lp(a) lowering compounds on Lp(a) and ASCVD events, and the potential role CAC scoring may have in defining the ideal patient for these novel therapies.

## Author contributions

All authors listed have made a substantial, direct, and intellectual contribution to the work and approved it for publication.

## References

[1] Grundy SM Stone NJ Bailey AL Beam C Birtcher KK Blumenthal RS. 2018 AHA/ACC/AACVPR/AAPA/ABC/ACPM/ADA/AGS/ APhA/ASPC/NLA/PCNA Guideline on the Management of Blood Cholesterol: a report of the American College of Cardiology/American Heart Association Task Force on Clinical Practice Guidelines. J Am Coll Cardiol. (2019) 73:e285–e350. 10.1016/j.jacc.2018.11.003 30423393

[2] Garg PK Guan W Karger AB Steffen BT Budoff M Tsai MY. Lipoprotein (a) and risk for calcification of the coronary arteries, mitral valve, and thoracic aorta: the Multi-Ethnic Study of Atherosclerosis. J Cardiovasc Comput Tomogr. (2021) 15:154–60. 10.1016/j.jcct.2020.06.002 32620506 PMC7750253

[3] Guerra R Yu Z Marcovina S Peshock R Cohen JC Hobbs HH. Lipoprotein (a) and apolipo-protein (a) isoforms: no association with coronary artery calcification in the Dallas Heart Study. Circulation. (2005) 111:1471–9. 10.1161/01.CIR.0000159263.50305.BD 15781743

[4] Kullo IJ Bailey KR Bielak LF Sheedy PF Klee GG Kardia SL. Lack of association between lipoprotein(a) and coronary artery calcification in the Genetic Epidemiology Network of Arteriopathy (GENOA) study. Mayo Clin Proc. (2004) 79:1258–63. 10.4065/79.10.1258 15473406

[5] Cainzos-Achirica M Bittencourt MS Osei AD Haque W Bhatt DL Blumenthal RS. Coronary artery calcium to improve the efficiency of randomized controlled trials in primary cardiovascular prevention. J Am Coll Cardiol Img. (2021) 14:1005–16. 10.1016/j.jcmg.2020.10.016 33221237

[6] Mehta A Vasquez N Ayers CR Patel J Hooda A Khera A. Independent association of lipoprotein(a) and coronary artery calcificationp with atherosclerotic cardiovascular risk. J Am Coll Cardiol. (2022) 79:757–68. 10.1016/j.jacc.2021.11.058 35210030 PMC10966924

[7] Greif M Arnoldt T von Ziegler F Ruemmler J Becker C Wakili R. Lipoprotein (a) is independently correlated with coronary artery calcification. Eur J Intern Med. (2013) 24:75–9. 10.1016/j.ejim.2012.08.014 23021791

[8] Pechlivanis S Mahabadi AA Hoffmann P Nöthen MM Broecker-Preuss M Erbel R. Association between lipoprotein(a) (Lp(a)) levels and Lp(a) genetic variants with coronary artery calcification. BMC Med Genet. (2020) 21:62. 10.1186/s12881-020-01003-3 32220223 PMC7099786

[9] Qasim AN Martin SS Mehta NN Wolfe ML Park J Schwartz S. Lipoprotein(a) is strongly associated with coronary artery calcification in type-2 diabetic women. Int J Cardiol. (2011) 150:17–21. 10.1016/j.ijcard.2010.02.021 20303190 PMC3132301

[10] Landray MJ Haynes R Hopewell JC Parish S Aung T Tomson J. Effects of extended-release niacin with laropiprant in high-risk patients. N Engl J Med. (2014) 371:203–12. 10.1056/NEJMoa1300955 25014686

[11] Boden WE Probstfield JL Anderson T Chaitman BR Desvignes-Nickens P Koprowicz K. Niacin in patients with low HDL cholesterol levels receiving intensive statin therapy. N Engl J Med. (2011) 365:2255–67. 10.1056/NEJMoa1107579 22085343

[12] O'Donoghue ML Fazio S Giugliano RP Stroes ESG Kanevsky E Gouni-Berthold I. Lipoprotein(a), PCSK9 inhibition, and cardiovascular risk. Circulation. (2019) 139:1483–92. 10.1161/CIRCULATIONAHA.118.037184 30586750

